# Prevalence of *Candida albicans* in the oral cavity of Beta Thalassemia Major and Thalassemia Minor Patients

**DOI:** 10.12688/f1000research.162460.1

**Published:** 2025-06-03

**Authors:** Saba Sami Abd Al Wahab, Maha Adel Mahmood

**Affiliations:** 1 Department of Basic Sciences, College of Dentistry, Baghdad University, Baghdad, Baghdad Governorate, Iraq

**Keywords:** Thalassemia Major, Thalassemia Minor, Candida albicans, Iron Overload, Ferritin levels

## Abstract

**Aims:**

To examine the correlation between iron, ferritin concentrations, and *C. albicans* infection in individuals with beta-thalassemia major and beta-thalassemia minor compared with healthy subjects.

**Materials and methods:**

It involved 90 participants, thirty patients with thalassemia major and thirty patients with thalassemia minor compared with thirty healthy controls. Saliva samples were obtained and cultivated to isolate, identify, and calculate the viable colony count of *C. albicans* in (cfu/ml). In contrast, serum levels of iron and ferritin were quantified using chemical analyzers.

**Result:**

Showed that 73.33% of thalassemia major group exhibited oral *C. albicans* colonization, which is significantly higher than that of thalassemia minor group 40% and control group 6.67%. Biochemical analyses revealed significantly higher iron 278.82 μg/dl and ferritin 2783.80 ng/ml levels in major group p<0.001 when compared with both thalassemia minor group 122.652 μg/dl, 74.723 ng/ml and control groups 127.438 μg/dl and 63.150 ng/ml respectively. *C. albicans* colony count in saliva was significantly elevated in beta-thalassemia major, as compared with beta-thalassemia minor group and control group.

**Conclusion:**

Findings suggest that iron overload, which results from recurrent blood transfusions and causes immune dysfunction, contributes to higher risk of oral fungal infections in Beta-thalassemia patients compared with controls.

## Introduction

Thalassemia is a hereditary condition affecting hemoglobin, the condition involves a decrease or absence in the formation of one or more globin chains in the hemoglobin tetramers, resulting in uncontrolled destruction of red blood cells and, ultimately, severe anemia. ^1, 2^ Beta-thalassemia arises from mutations that impact many stages of beta-globin protein synthesis, encompassing transcription, translation, and the stability of beta-globin production. ^3^ One of the most prevalent genetic illnesses in humans is β-thalassemia. Around 60,000 infants worldwide are born with β-thalassemia annually, predominantly in countries of the Mediterranean but also in the Middle East, West Africa, India, and South-East Asia. ^4^ The estimated prevalence of thalassemia carriers is 3 to 10 individuals per 100 individuals. Given a population of 200 million individuals, a birth rate of 20%, and a thalassemia carrier rate of around five percent, it is projected that there will be an annual occurrence of 2,500 kids born with thalassemia congenital illness. ^5^ Patients with β-thalassemia often have blood transfusions and suffer from iron overload. ^6^ Serum ferritin (SF) indicates probable iron excess. ^7^ Serum ferritin is an effective monitoring instrument for iron overload in thalassemia major. ^8^ In healthy individuals, the average serum ferritin (SF) levels range from 12 to 300 μg/L for men and from 12 to 150 μg/L for females. ^9^ Serum ferritin levels exceeding 1000 μg/L are indicative of iron overload and are linked to adverse outcomes, including increased mortality and organ injury, a more significant likelihood of cardiac events, and hepatic difficulties. ^10^ *Candida albicans* is a fungal infection that results in approximately 1.7 million fatalities annually on a global scale, primarily affecting those with weakened immune systems and several underlying medical problems. ^11^ The prevalence of infectious illnesses by Candida has consistently risen since the 1970s, potentially attributed to an elevated susceptibility to opportunistic infections, advancements in clinical techniques for detecting fungi-related hospital-acquired infections, and the emergence of antifungal resistance resulting from prolonged treatment exposure. ^12, 13^ The clinical features of oral cavities in patients with thalassemia include pointed and shortened root morphology, taurodontism, and a characteristic chipmunk appearance. These individuals often have a higher caries index and display gingival hypertrophy, which suggests gingival inflammation. Additionally, these patients are susceptible to infections caused by bacteria or fungi, including *C. albicans.* ^14^ Thalassemia, especially thalassemia major, is linked to a heightened incidence of *C. albicans* colonization in the oral cavity. This increased vulnerability is chiefly attributable to variables including iron accumulation from repeated blood transfusions, which creates a nutrient-rich environment favorable for fungal proliferation, and immunological impairment caused by the disease and its therapies. ^15^ This study aims to examine the correlation between iron and ferritin levels and *C. albicans* distribution in individuals with major and minor beta-thalassemia compared with healthy controls.

## Methods

### Subject selection

The saliva and blood samples were collected between December 2023 and March 2024 from a total of sixty patients; thirty of them were individuals diagnosed with Beta Thalassemia Major, and thirty were diagnosed with Beta Thalassemia Minor attending the Thalassemia Centers of Ebn-Albalidy and Al-Karama hospitals in Baghdad compared with thirty apparently healthy individuals served as control. Data collected from each participant included demographic information (name, age, gender) and duration of illness. The Research Ethics Committee of the University of Baghdad College of Dentistry granted ethical permission for the study, with reference number 889 and project number 889824, on January 11, 2024. The research was conducted in conformity with the principles established in the Declaration of Helsinki. Before doing the analysis, all patient data was anonymized.

### Inclusion criteria

Inclusion criteria included patients with stable Beta Thalassemia Major (T Major) without other diseases or medication influences this group representing the 1 ^st^ patients group, and stable Beta Thalassemia minor (T Minor) patients with no significant health changes, representing the 2 ^nd^ patients group compared with apparently healthy individuals with no blood disorders or chronic illnesses affecting iron metabolism representing the control group. All participants with ages ranged between 18-60 years.

### Exclusion criteria

Exclusion criteria included any subject between 18 and 60 years of age but with an inability to provide informed consent or with conditions or treatments that could affect the biomarkers being studied. Indeed, pregnant or lactation women were also excluded.

### Serum and saliva collection

Three milliliters of unstimulated saliva were obtained from each participant by the spitting method, ^16^ and subsequently transferred to sterile tubes. The samples were centrifuged at 4000 rpm for 3 minutes to separate cellular debris. For blood samples, 5 mL were obtained through venipuncture into sterile tubes, centrifuged for 15 minutes at 3000 rpm, and the serum extracted using an automated pipette. Both serum and saliva were preserved at -20°C until later usage.

### Isolation and counting of *Candida albicans*

Saliva samples from both patients and control groups were processed using a serial dilution method ^17^ 4.5 ml of nutrient broth was added to sterile tubes, and 0.5 ml of freshly collected saliva was introduced into the first tube, followed by inoculating the following tubes successively to achieve the tenfold dilutions. One hundred microliters of each dilution were distributed over a plate of Sabouraud Dextrose Agar (SDA) medium containing 1% chloramphenicol and incubated at 37°C for 24-48 hours. The resulting colony count were determined by CFU/ml.

### Microscopic identification of *Candida albicans*

*C. albicans* were initially identified and explored microscopically to observe the oval or elongated yeast cells exhibiting budding and pseudohyphae. Additionally, the *C. albicans* inoculum was incubated in 3-5 ml of human serum. A small quantity of the grown serum was placed on a glass slide and examined under a microscope to determine the presence or absence of germ tube formation.

### Identification by using chromogenic test and VITEK- 2 compact system

*C. albicans* were subcultured onto a chromogenic medium (CHRO Magar TM Candida media) and then incubated at 37°C for 48 to 72 hours. This technique uses substrates linked to chemical dyes to distinguish several species of Candida by the coloration of the developing colonies. For additional identification, the VITEK ^®^ 2 COMPACT was employed to accurately identify *C. albicans* isolates to the species level, according to the manufacturer’s guidelines (Biomerieux/France).

### Measurement of serum iron and ferritin levels by chemistry analyzers

The method employs microparticle-enhanced immunoturbidimetry using a Thermo Scientific™ Indiko™ Clinical Chemistry Analyze (Finland). Sera samples from patients and controls were utilized and processed with microparticles coated with rabbit antibodies specific to either human ferritin or iron. The rabbit antibodies specific to human ferritin and iron were not sourced directly from live animal cells in our laboratory. They were obtained externally as part of commercial reagent kits from Thermo Scientific: the Human Ferritin Chemistry Analyzer Kit (Ref 981949, 4 × 4 mL, Indiko™) and the Human Iron Chemistry Analyzer Kit (Ref 981236, 10 × 20 mL, Konelab™, Indiko™). These kits provide pre-coated microparticles with polyclonal rabbit anti-human antibodies, validated for use with the Thermo Scientific™ Indiko™ Clinical Chemistry Analyzer. The measurement of immunocomplex formation for Ferritin is conducted by assessing absorbance variations at 700 nm, within a detection range of 0.32–20 ng/mL, iron immunocomplex formation is quantified by absorbance at 600 nm.

### Statistical analysis

The statistical analyses were carried out by applying the SPSS 15 software (SPSS Inc., IL, USA, https://spssdownload.com/spss-software-version-15-free-download). It was employed the Shapiro-Wilk test, Kruskal-Wallis H test, and the Pairwise test. The p-value of less than 0.05 was considered statistically significant.

## Result

The research study examined the age-based disparities in mean values for the T Major and T Minor groups and for the control group. T Minor group demonstrated a superior mean value of age (35.533 ± 7.523) years, followed by the T Major group (23.233 ± 7.398) years in comparison with the control group as it had a somewhat elevated mean than the minor group (29.667 ± 9.517). Table 1.

**Table 1.** Age-specific distribution of mean ± standard deviation values among groups.

	Control		T Major		T Minor	
	N	Mean ± S.D.	N	Mean± S.D.	N	Mean ± S.D.
Total	30	29.667 ± 9.517	30	23.233 ± 7.398	30	35.533 ± 7.523

### Isolation and identification of *Candida albicans*

**Figure 1.**
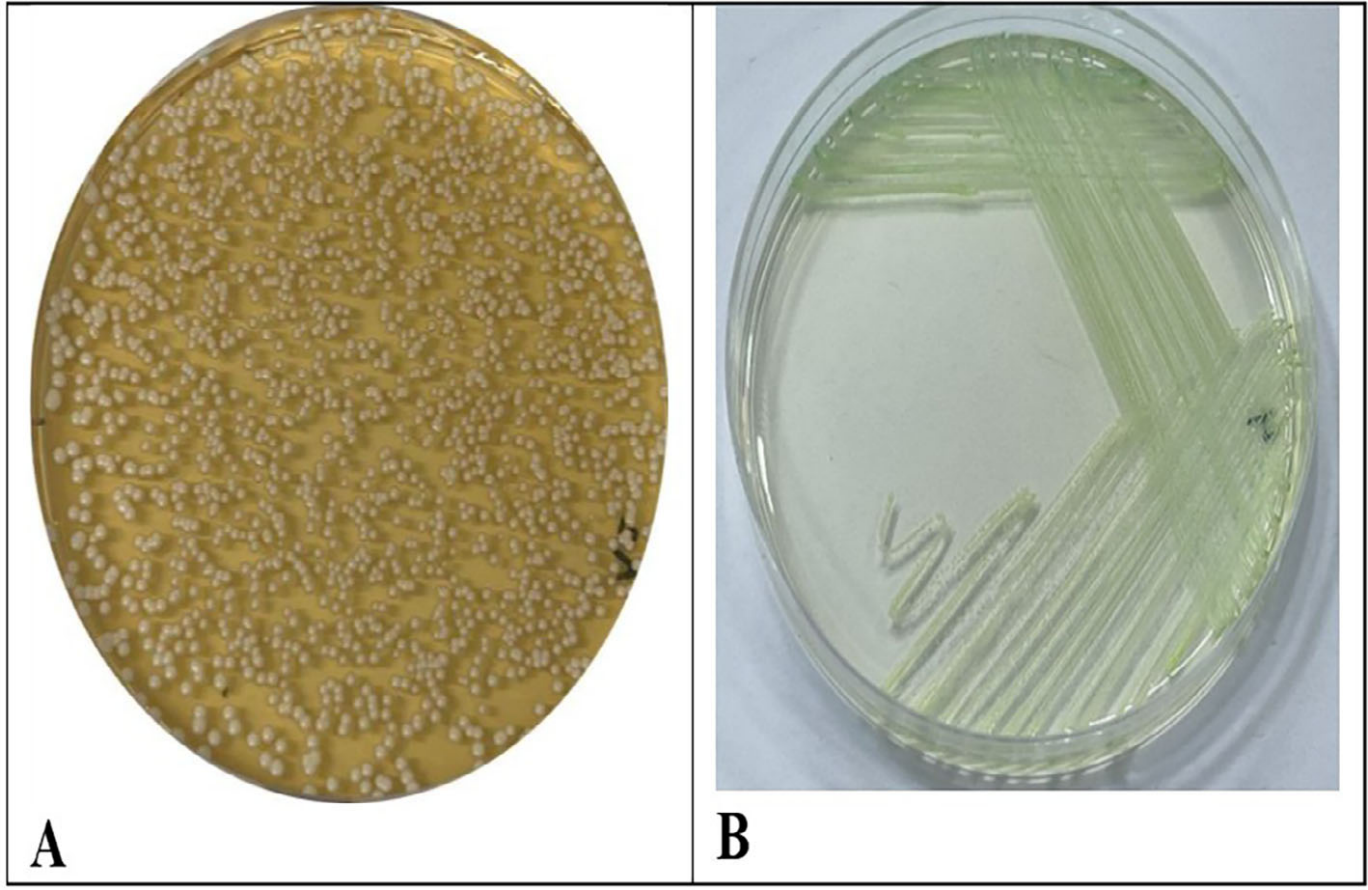
(A) Shows colonies of *C. albicans* on SDA (B) Shows *C. albicans* on CHRO Magar.

*C. albicans* isolates were isolated and diagnosed successfully from the saliva of patients suffering from B-thalassemia. *C. albicans* display distinctive colonies. When cultivated on (SDA), the colonies exhibited a smooth, creamy texture and were white to off-white in hue. They are often grown with a delicate texture. Over time, the colonies may display the development of a corrugated surface Figure 1. CHRO Magar was utilized for more accurate identification. In this instance, colonies of *C. albicans* display a characteristic green coloration due to the enzymatic activity of the yeast. The combination of these characteristics with microscopic identification facilitates the accurate diagnosis of *C. albicans* infections.

**Figure 2.**
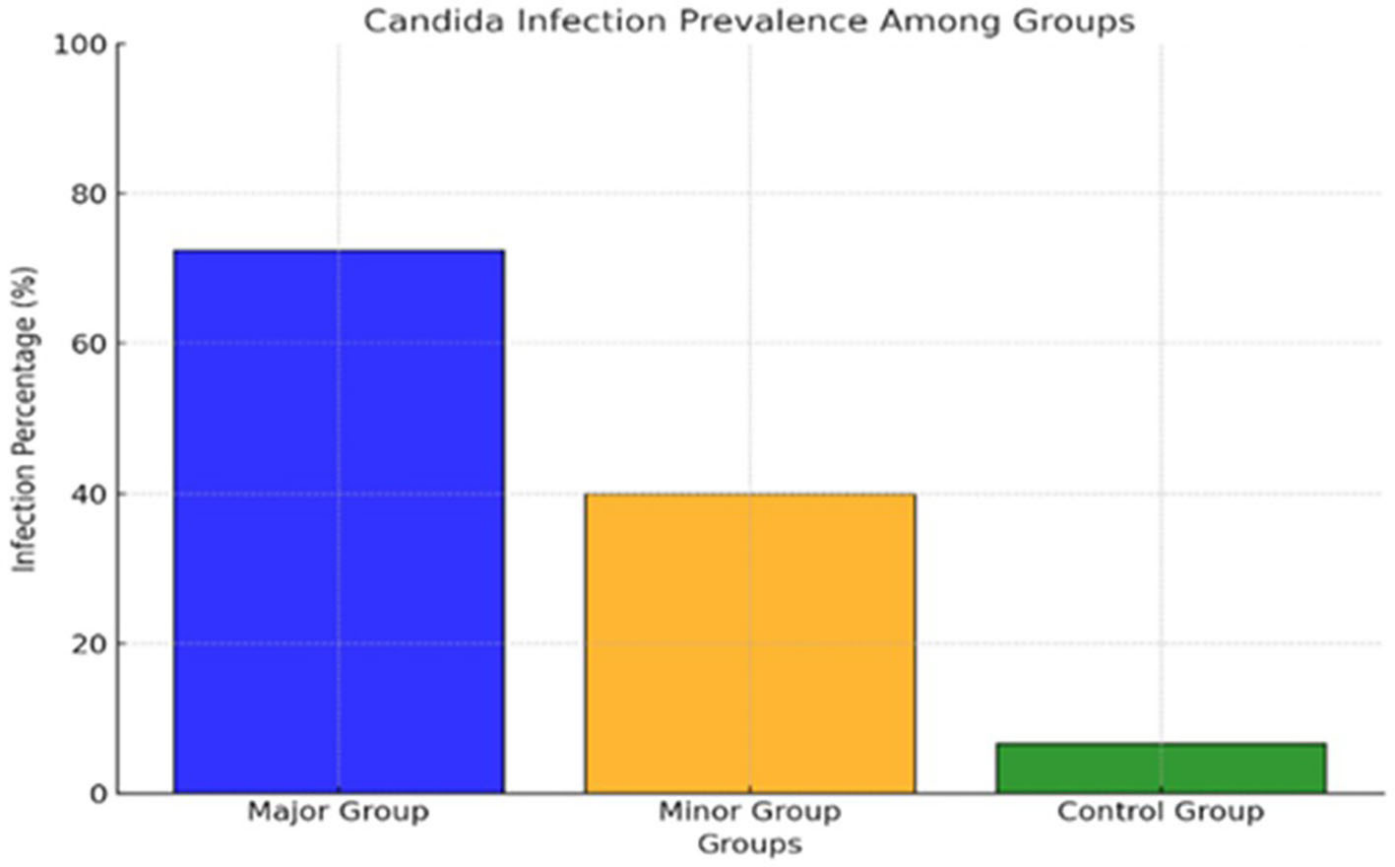
The distribution of salivary *C. albicans* among groups.

For advanced identification, all isolates were accurately identified using the VITEK 2 Compact System, with a probability of 88% to 90%, hence demonstrating the system’s efficiency in identifying *C. albicans.* Figure 2 and Table 2 show the distribution of *C. albicans* among the groups as the T Major has the highest carriage rate, as twenty-two out of thirty patients have *C. albicans* accounting for (73.33%) of the group. In contrast, in the T Minor, only twelve out of thirty patients carrying *C. albicans* represented (40%) of the group, and the control group exhibited the lowest prevalence rate with merely two individuals, constituting (6.67%) of the healthy subjects. The results indicated a markedly elevated colonization rate of *C. albicans* in the major group relative to the minor and control groups, respectively.

**Table 2.** Distribution of *C. albicans* among groups.

	T Major		T Minor		Control	
	Present (%)	Absent	Present	Absent	Present	Absent
No. Total	22(73.33)	8	12(40)	18	2(6.67)	28

### Measurement of Iron and ferritin levels in B-thalassemia patients

This study analyzed two biochemical parameters, Iron and ferritin, across the beta thalassemic Major and Minor patients in comparison with the control group. Descriptive statistics, comprising the median, which was computed for each parameter simultaneously with *C. albicans.* Statistical analysis indicated substantial differences across the groups (p < 0.001) for all parameters, as the T-Major group revealed a higher iron, ferritin, and *C. albicans* count when compared with the results of both T Minor and control groups, as seen in Table 3.

**Table 3.** Descriptive statistics for iron, ferritin, and *C. albicans* levels among study groups.

Parameters	Groups	Descriptive statistics		
		Median	X ^2^	P-value
Iron (μg/dl)	T Major	270.016	58.919	0.000
	T Minor	117.599		
	Control	116.985		
Ferritin (mg/ml)	T Major	2783.800	59.376	0.000
	T Minor	77.800		
	Control	63.150		
*C. albicans* (x10 ^4^) cfu/ml	T Major	1.715	28.068	0.000
	T Minor	0		
	Control	0		

The T Major group for Iron demonstrated the most excellent median (270.016) in comparison to the Control (116.985) and T Minor (117.599). Likewise, for Ferritin, T Major had a significantly higher median (2783.800), greatly surpassing T Minor (77.800) and control (63.150). In addition to these biochemical markers, *C. albicans* levels in saliva were significantly elevated in the T Major group (1.715 x10 ^4^ cfu/ml) compared to the Minor and control as the median has zero value for both groups. These data demonstrate substantial increases in these parameters within the T Major group vs. other groups.

The results in Table 4 illustrated the pairwise analysis of Iron, Ferritin, and *C. albicans* levels among the T Major, T Minor and control groups demonstrated substantial increases in the T Major group relative to the control group for all three parameters (p < 0.001). Likewise, significant differences were noted between the T Major and T Minor groups across all parameters (p ≤ 0.005), further emphasizing the heightened levels in the T Major group. No significant differences were observed between the T Minor and control groups for the biochemical parameters, Iron and Ferritin (p = 0.748 and p = 0.851, respectively), and the difference for *C. albicans* was also not statistically significant between these groups (p = 0.106).

**Table 4.** Pairwise comparisons and P-values for iron, ferritin, and *Candida albicans* concentrations across study cohorts.

Parameters	Groups		Pairwise test	p-value
Iron (μg/dl)	Control	T Major	-43.717	0.000
		T Minor	2.167	0.748
	T Major	T Minor	45.883	0.000
Ferritin (mg/ml)	Control	T Major	-44.367	0.000
		T Minor	1.267	0.851
	T Major	T Minor	45.633	0.000
*C. albicans*	Control	T Major	-31.433	0.000
		T Minor	-12.567	0.106
	T Major	T Minor	18.867	0.005

### Correlation between markers for study groups

The results in Tables 5, 6, and 7 showed no significant correlations among all markers under investigation (iron, ferritin, and *C. albicans* levels) in both patient groups (T Major and T Minor) and the control group, except for a significant correlation noted between iron and ferritin levels in the control group.

**Table 5.** Correlation between markers in T Major group.

Parameters		C. albicans	Ferritin
Iron (μg/dl)	r	-0.062	0.092
	p	0.746	0.628
Ferritin (mg/ml)	r	-0.214	
	p	0.257	

**Table 6.** Correlation between markers in the T Minor group.

Parameters		C. albicans	Ferritin
Iron (μg/dl)	r	0.082	0.343
	p	0.668	0.064
Ferritin (mg/ml)	r	0.037	
	p	0.844	

**Table 7.** Correlation between markers in the control group.

Parameters		C. albicans	Ferritin
Iron (μg/dl)	r	0.040	0.499
	p	0.833	0.005
Ferritin (mg/ml)	r	0.065	
	p	0.734	

## Discussion

The result of this study indicated that 73.33% of the significant thalassemia patient group had oral cavity colonization by *C. albicans*, a substantially greater rate compared to the minor group at 40% when compared with the control group which displayed the lowest prevalence at 6.67%. A markedly elevated mean candidal count was noted in thalassemic patients in comparison to the healthy cohort, the findings closely agree with another study conducted in Jordan, which indicated that Candida species were identified in 74% of thalassemic patients. Similarly, the present results obtained were comparable to another study conducted in Egypt, *as C. albicans* was isolated in 69.2% of cases, whereas 30.8% were non- *C. albicans.* ^18^ The data obtained exhibited a higher elevation rate of *C. albicans* when compared with a local study performed in Najaf, Iraq, which evaluated samples from 50 thalassemia patients, of which only 14(28%) demonstrated positive growth for Candida species. ^19^ One of the primary reasons that significant *C. albicans* have infected some minor patients is the immune problems that may have increased sensitivity to oral fungal colonization in these thalassemic patients. ^20^ This fact may be due to the deficiency in neutrophils and macrophage chemotaxis, which have been documented in thalassemic individuals, especially in those experiencing iron overload. ^21^ Anemia may serve as a predisposing factor for oral fungal colonization in people with thalassemia. ^22^ Another reason is that repeated blood transfusions lead to iron accumulation in salivary glands, resulting in the development of non-transferrin-bound iron (NTBI), which circulates in plasma and generates reactive oxygen species (ROS). ^23^ Iron accumulation in salivary gland acini cells induces inflammation and reduces salivary output and components. ^24^ Salivary components bolster the immune system’s response to infections caused by *C. albicans* by preventing the spread and protecting the mucosal epithelial barrier. Reduced salivary production in beta-thalassemia major patients can lead to dysbiosis, causing *C. albicans* to overgrow and adhere to the oral epithelium. ^25^ Increased levels of iron and ferritin create an environment favorable for the growth of *C. albicans* in the major group. ^26^ The findings of the current study reveal a unique biochemical and microbiological profile in the T Major group, marked by considerably increased levels of iron, ferritin, and *C. albicans* as compared to the T Minor and control groups. The T Major group exhibited significantly elevated iron and ferritin levels, indicating modified iron metabolism, potentially associated with inflammation or stress responses, alongside markedly increased *C. albicans* concentrations, indicating possible microbiome disruption or immune system changes. The pairwise analysis confirmed that these elevations were statistically significant (p < 0.001), differentiating the T Major group, from each of minor and control group. Still, no significant changes were noted between the T Minor and control groups for iron and ferritin (p = 0.748 and p = 0.851) or *C. albicans* (p = 0.106). The results obtained correspond with another two previous studies, one conducted in Turkey and the other in Indonesia. Both studies investigated iron and ferritin levels in saliva and serum across various groups, including thalassemia patients (major and minor) and controls. The findings of these studies indicate significantly elevated blood ferritin and iron levels in the significant thalassemia group compared to both minor and control groups. ^27, 28^ The findings of the current investigation align with those of a study performed in Pakistan involving 155 patients with T Major. The study also revealed markedly increased ferritin and iron levels in the T Major group relative to the control group. ^29^ The increased concentrations of iron and ferritin in individuals in the T Major group are mainly due to the frequent blood transfusions necessary for the treatment of severe anemia. Each transfusion sends roughly 200 mg of iron into the body, ^30^ and due to the absence of a natural physiological mechanism for excreting excess iron, patients progressively collect iron. ^31^ This iron overload results in the accumulation of surplus iron in tissues and organs, evidenced by markedly increased serum ferritin levels, a crucial indicator of iron storage. ^32^ Moreover, the inadequate erythropoiesis in beta-thalassemia significantly leads to heightened intestinal iron absorption, hence intensifying iron buildup. In the absence of sufficient chelation therapy, iron excess may escalate to dangerous levels, leading to problems such as organ damage. ^33^ Conversely, beta-thalassemia minor is marked by less severe anemia as it resulting from a singular faulty beta-globin gene. ^34^ Individuals with this illness generally do not necessitate blood transfusions, hence reducing the risk of iron excess from exogenous sources. ^35^ Moreover, the extent of inefficient erythropoiesis is considerably reduced in beta-thalassemia minor, resulting in a better-controlled iron absorption and storage mechanism. As a result, their iron and ferritin levels typically stay within normal limits. ^36^ The present work illustrated a correlation between biochemical markers and colonization by *Candida albicans* in thalassemia patients. The increased levels of *C. albicans* in individuals in the T Major group may be indirectly associated with the raised concentrations of iron and ferritin in this population. ^21^ Iron is essential for microbial proliferation and pathogenicity, particularly in fungal infections such as *C. albicans.* ^37^ In instances of iron overload, such as in beta-thalassemia major, excess free iron in the bloodstream and tissues could be served as a plentiful substrate for Candida, facilitating its growth. ^38^ Ferritin, a principal iron storage protein, is also raised in beta-thalassemia major as a result of continuous transfusions and poor erythropoiesis. ^39, 40^ Candida might have evolved ways to extract iron from host ferritin, hence augmenting its survival and proliferation in iron-abundant settings. Conversely, patients with beta-thalassemia minor demonstrate normal or nearly normal iron and ferritin levels owing to the lack of recurrent transfusions and modest inefficient erythropoiesis. ^41^ As a result, there is an absence of surplus iron to promote Candida proliferation, and their immune systems are often less impaired. This leads to markedly reduced amounts of *C. albicans* in patients with beta-thalassemia minor relative to those with beta-thalassemia major. ^42^

## Conclusion

This study shows the heightened prevalence of *C. albicans* colonization in patients with thalassemia major relative to individuals with thalassemia minor and healthy controls. The results indicate that iron overload, diminished immune system impairment, and contribute to the increased vulnerability to fungal infections in thalassemia major patients. These findings underscore the significance of iron control and iron chelation therapy in averting problems, such as oral fungal infections, and indicate that consistent monitoring may enhance patient outcomes.

### Ethics and consent

The Research Ethics Committee of the University of Baghdad College of Dentistry granted ethical permission for the study, with reference number 889 and project number 889824, on January 11, 2024. The study started on February 1, 2024, and was conducted in conformity with principles established in the Declaration of Helsinki. Before doing the analysis, all patient data was anonymized.

All participants provided written informed consent, as approved by the Research Ethics Committee of the University of Baghdad, College of Dentistry, which granted ethical permission for the study. The patients were fully informed about the study’s objectives, procedures, and potential benefits, and were assured of their complete freedom to participate. Consent was obtained for the collection of both blood and saliva samples.

## Data Availability

Figshare: my data.xlsx. https://doi.org/10.6084/m9.figshare.28399424.v1. ^43^ The project contains the following underlying data:
• my data.xlsx Data are available under the terms of the Creative Commons Attribution International license (CC BY 4.0).
