# Supplementary material for: Prevalence of Candida albicans in the oral cavity of Beta Thalassemia Major and Thalassemia Minor Patients
Source: F1000Res. 2025 Aug 21;14:555. Originally published 2025 Jun 3. [Version 2] doi: 10.12688/f1000research.162460.2 (PMC12351238; doi:10.12688/f1000research.162460.2)
Supplement: Peer review history

# Reviewer report: Reviewer response for version 2

**DOI:** 10.5256/f1000research.186768.r407571

**Published:** 2025-08-22

**Authors:** Anwer Faisal

**Affiliations:** 1 Mustansiriyah University, Baghdad, Iraq

I have reviewed the authors' revisions and the summary of changes made in response to the peer review reports. The authors have adequately addressed all the required modifications and concerns raised during the review process.

 I confirm my **Approved** status for this revised version. The improvements made strengthen the manuscript and I believe it is now ready for indexing.

Is the work clearly and accurately presented and does it cite the current literature?

Yes

If applicable, is the statistical analysis and its interpretation appropriate?

Yes

Are all the source data underlying the results available to ensure full reproducibility?

Yes

Is the study design appropriate and is the work technically sound?

Partly

Are the conclusions drawn adequately supported by the results?

Yes

Are sufficient details of methods and analysis provided to allow replication by others?

Yes

Reviewer Expertise:

Microbiology - pathogenic bacteria

I confirm that I have read this submission and believe that I have an appropriate level of expertise to confirm that it is of an acceptable scientific standard.

# Reviewer report: Reviewer response for version 1

**DOI:** 10.5256/f1000research.178665.r392347

**Published:** 2025-08-13

**Authors:** Pradana Zaky Romadhon

**Affiliations:** 1 Airlangga University, Surabaya, Indonesia

The submitted article is quite interesting and has been written with good structure and clarity. However, there are several point that might enhance the article, which we outline as follows:

- *Candida albicans* levels, which were also assessed as quantitative data, are not described in the Methods section. The authors should include a clear description of the methodology used to obtain quantitative data for *C. albicans*.
- In the Discussion section, a brief explanation should be provided regarding the mechanism by which iron overload impairs the immune system, making thalassemia patients more susceptible to *Candida albicans* infections.
- The authors are also encouraged to elaborate on the limitations that may have contributed to the lack of significant correlation among the investigated markers (iron, ferritin, and *C. albicans* levels) across the T major, T minor, and control groups.

Is the work clearly and accurately presented and does it cite the current literature?

Yes

If applicable, is the statistical analysis and its interpretation appropriate?

I cannot comment. A qualified statistician is required.

Are all the source data underlying the results available to ensure full reproducibility?

Yes

Is the study design appropriate and is the work technically sound?

Yes

Are the conclusions drawn adequately supported by the results?

Yes

Are sufficient details of methods and analysis provided to allow replication by others?

Partly

Reviewer Expertise:

Thalasemia and Hemophilia

I confirm that I have read this submission and believe that I have an appropriate level of expertise to confirm that it is of an acceptable scientific standard.

# Reviewer report: Reviewer response for version 1

**DOI:** 10.5256/f1000research.178665.r389913

**Published:** 2025-06-13

**Authors:** Anwer Faisal

**Affiliations:** 1 Mustansiriyah University, Baghdad, Iraq

This well-designed study provides valuable clinical insights into how iron overload may impact Candida infections in thalassemia patients. By comparing three distinct groups - those with major and minor forms of the disease alongside healthy controls - the researchers uncovered striking differences in fungal colonization rates that clearly track with disease severity. The finding that over 70% of major thalassemia patients carried oral Candida, compared to just 6% of healthy individuals, is particularly noteworthy for clinicians managing these cases.

 While the laboratory methods appear sound and the statistical approach appropriate, we did note that the study could have been strengthened by including more details about how thalassemia was diagnosed in participants. A simple mention of whether genetic testing or hemoglobin electrophoresis was used would help readers better assess the accuracy of patient grouping.

 Despite this minor limitation, the research makes a compelling case for the connection between iron overload and fungal susceptibility in this vulnerable population.

Is the work clearly and accurately presented and does it cite the current literature?

Yes

If applicable, is the statistical analysis and its interpretation appropriate?

Yes

Are all the source data underlying the results available to ensure full reproducibility?

Yes

Is the study design appropriate and is the work technically sound?

Partly

Are the conclusions drawn adequately supported by the results?

Yes

Are sufficient details of methods and analysis provided to allow replication by others?

Yes

Reviewer Expertise:

Microbiology - pathogenic bacteria

I confirm that I have read this submission and believe that I have an appropriate level of expertise to confirm that it is of an acceptable scientific standard.
